# The multi-site docking protein Grb2-associated binder 1 (Gab1) enhances interleukin-6-induced MAPK-pathway activation in an SHP2-, Grb2-, and time-dependent manner

**DOI:** 10.1186/s12964-019-0451-2

**Published:** 2019-10-24

**Authors:** Hannes Bongartz, Karen Gille, Wiebke Hessenkemper, Katharina Mandel, Marc Lewitzky, Stephan M. Feller, Fred Schaper

**Affiliations:** 1 Institute of Biology, Department of Systems Biology, Otto-von-Guericke University, Universitätsplatz 2, Gebäude 28/Pfälzer Platz, 39106 Magdeburg, Germany; 2 Institute of Molecular Medicine, Charles Tanford Protein Research Center, Martin-Luther-University Halle-Wittenberg, Kurt-Mothes-Straße 3a, 06120 Halle (Saale), Germany

**Keywords:** Interleukin-6, IL-6, Janus kinase, Jak, Gab1, SHP2, PI3K, MAPK, Erk, c-Fos, STAT, Signal transduction, Signal orchestration, Cytokines

## Abstract

**Background:**

Cytokine-dependent activation of signalling pathways is tightly orchestrated. The spatiotemporal activation of signalling pathways dictates the specific physiological responses to cytokines. Dysregulated signalling accounts for neoplastic, developmental, and inflammatory diseases. Grb2-associated binder (Gab) family proteins are multi-site docking proteins, which expand cytokine-induced signal transduction in a spatial- and time-dependent manner by coordinating the recruitment of proteins involved in mitogen activated protein kinase (MAPK)/extracellular-signal regulated kinase (ERK) and phosphatidyl-inositol-3-kinase (PI3K) signalling. Interaction of Gab family proteins with these signalling proteins determines strength, duration and localization of active signalling cascades. However, the underlying molecular mechanisms of signal orchestration by Gab family proteins in IL-6-induced signalling are only scarcely understood.

**Methods:**

We performed kinetic analyses of interleukin-6 (IL-6)-induced MAPK activation and analysed downstream responses. We compared signalling in wild-type cells, Gab1 knock-out cells, those reconstituted to express Gab1 mutants, and cells expressing gp130 receptors or receptor mutants.

**Results:**

Interleukin-6-induced MAPK pathway activation can be sub-divided into an early Gab1-independent and a subsequent Gab1-dependent phase. Early Gab1-independent MAPK activation is critical for the subsequent initiation of Gab1-dependent amplification of MAPK pathway activation and requires binding of SH2 domain-containing phosphatase 2 (SHP2) to the interleukin-6 receptor complex. Subsequent and coordinated recruitment of Grb2 and SHP2 to Gab1 is essential for Gab1-dependent amplification of IL-6-induced late MAPK pathway activation and subsequent gene expression.

**Conclusions:**

Overall, we elaborated the molecular requirements for Gab1-dependent, spatiotemporal orchestration of interleukin-6-dependent MAPK signalling. We discriminated IL-6-induced Gab1-independent, early activation of MAPK signalling and Gab1-dependent, sustained activation of MAPK signalling.

## Plain English summary

The cytokine interleukin-6 (IL-6) is a prominent tissue hormone that regulates the inflammatory response. Stringent and well controlled action of IL-6 function is crucial because malregulated IL-6 signalling contributes to inflammatory and autoimmune diseases and cancer. IL-6 activates signalling pathways inside the cell to trigger specific cellular responses. One of these pathways is the so called mitogen-activated protein kinase (MAPK) pathway. The duration and strength of MAPK activation in the cell determines the specific response of the cell. In this study, we elaborated the impact of the protein Gab1 which orchestrates MAPK activation. We found that early and transient MAPK activation is Gab1 independent, whereas sustained activation of MAPK signalling requires Gab1. Furthermore, we elucidated the molecular mechanisms of Gab1 action.

## Background

Ligand-induced activation of cytokine receptors leads to subsequent activation of intracellular signalling cascades. One important step to induce signalling cascades by cytokines is the phosphorylation of tyrosine residues in the cytoplasmic part of activated cytokine receptors. The subsequent recruitment of signalling components to specific phosphorylated tyrosine motifs is a prerequisite for further activation of these components by phosphorylation, translocation and/or conformational changes. Multi-site adapter proteins contribute to signal processing by serving as docking platforms for a variety of specific signalling proteins. On the one hand, these signalling platforms contribute to the activation of signalling. On the other hand, they enable both amplified and sustained signalling and mutual regulation of signalling cascades. Thus, multi-site adapter proteins facilitate signal orchestration and thus highly impact on cytokine-induced cell fates.

Interleukin-6 (IL-6) is a pleiotropic cytokine and is involved in haematopoiesis, proliferation of plasma cells, and differentiation of leukocytes. IL-6 also induces the acute-phase response in hepatocytes. Therefore, IL-6 is strongly involved in the immune response (for reviews see [1–3]). IL-6 initiates the assembly of the IL-6-receptor complex by binding to the IL-6-receptor α (IL-6Rα). Subsequently, the IL-6:IL-6Rα complex recruits the signal transducing subunit glycoprotein 130 (gp130). Cells which do not express IL-6Rα can be stimulated with IL-6 in complex with soluble IL-6Rα (sIL-6Rα). At the fully assembled receptor complex, the Janus kinase (Jak)/signal transducer and activator of transcription (STAT) pathway is initiated. Additionally, STAT-independent signalling modules, such as the mitogen-activated protein kinase (MAPK) and the phosphatidylinositol-3-kinase (PI3K) cascade are also activated [1]. MAPK-cascade activation in response to IL-6 depends essentially on the recruitment of SH2-domain containing protein tyrosine phosphatase 2 (SHP2) to phosphorylated Y759 in the cytoplasmic region of gp130 [4].

Similar to the cytokine receptors, multi-site adapter proteins are also tyrosine phosphorylated in response to cytokine stimulation. One family of these scaffolding proteins is the Grb2-associated binder (Gab) family of the multi-site docking proteins. As suggested by their name, Gab proteins are constitutively associated with Grb2. Further, Gab proteins recruit signalling components, such as PI3K, SHP2, phospholipase C (PLC), or Ras-GTPase-activating protein (RasGAP). These proteins interact with Gab1 through specific phosphotyrosine motifs within the Gab protein. The resulting manifold interactions enable Gab family proteins to serve as signal computation modules in growth-factor and cytokine-induced signalling at the plasma membrane (for review see [5]).

Gab family proteins are recruited to the plasma membrane either by binding of their PH domain to phosphatidylinositol-3,4,5-trisphosphate (PIP3) or by binding to the cytoplasmic part of transmembrane receptors. Gab1 binds directly to the hepatocyte growth factor (HGF) receptor c-MET through its MET binding domain (MBD) [6]. Binding of Gab1 to the epidermal growth factor (EGF) receptor occurs via Grb2 [7].

Own studies revealed a more detailed picture of the molecular mechanisms of Gab1 membrane recruitment: under cytokine-deprived conditions, the PH domain of Gab1 is not accessible for PIP3 due to an intramolecular interaction of the PH domain with an peptide motif surrounding serine 552 of Gab1 [8, 9]. Release of this intramolecular blockade of the PH domain and subsequent binding of Gab1 to PIP3 at the plasma membrane depends on Erk1/2-induced phosphorylation of Gab1 at serine 552 [10]. Gab1 membrane recruitment and phosphorylation is malregulated in human erythroleukaemia cells, expressing the constitutive active JAK2-V617F mutant [11]. In line with this, uncontrolled proliferation of human erythroleukaemia cells depends on the expression of Gab1 [12].

Here, we show that the early MAPK pathway activation in response to IL-6 signalling is receptor-dependent, but Gab1-independent. This early MAPK activation enables Gab1 membrane translocation and phosphorylation and is a prerequisite for proper, sustained, and Gab1-dependent MAPK activation and subsequent c-Fos gene expression. Furthermore, interaction of both SHP2 and Grb2 with Gab1 are crucial for late Gab1-dependent MAPK activation in IL-6-induced signal transduction. We reveal a two-phase model consisting of an initial receptor-mediated activation and a subsequent, Gab1-dependent amplification of IL-6-induced MAPK-pathway activation.

## Materials and methods

### Materials

Recombinant human erythropoietin was purchased from Janssen (Germany). Hy-IL-6 was purchased from Conaris (Kiel, Germay). IL-5 was from Peprotech (Hamburg, Germany). The activation/phosphorylation-specific antibodies for tyrosine 627-phosphorylated Gab1 (#3233), threonine 202- and tyrosine 204-phosphorylated Erk1/2 (4370), tyrosine 705-phosphorylated STAT3 (#9131), tyrosine 524-phosphorylated SHP2 (#3751), serine 473-phosphorylated Akt (#4060), Akt (#4685), and the antibodies specific for Erk1/2 (#4695), and STAT3 (#9139) were obtained from Cell Signaling Technology (Germany). Gab1 recognizing antibody (#06–579) was bought from Millipore (USA). Antibody against SHP2 (#sc-280) was purchased from Santa Cruz (USA). GFP antibody (#600–101-215) was acquired from Rockland (USA). Antibody against phosphorylated serine 552 in Gab1 was prepared by immunization of rabbits with the corresponding phosphorylated peptide LQAPVR(p) SPITRSF coupled to KLH (Eurogentec, Belgium). Antibody against α-Tubulin (#T5168) was purchased from Sigma-Aldrich (USA). MEK inhibitor U0126 (Cell Signaling Technology, USA) and PI3K inhibitor Wortmannin (Calbiochem, USA) were dissolved in DMSO (Roth, Germany). Dulbecco’s modified Eagle’s medium (DMEM), Opti-MEM, zeocin, and hygromycin B were from Gibco Life Technologies (Germany). Fetal calf serum (FCS) was purchased from GE Healthcare (Germany). Puromycin and blasticidin were purchased from Roth (Germany).

### Expression vectors

Expression vectors for the IL-5R/gp130 receptor chimeras pRcCMV-IL-5Rα/gp130(YFYYYY), pRcCMV-IL-5Rβ/gp130(YFYYYY), pRcCMV-IL-5Rα/gp130(YYFFFF), pRcCMV-IL-5Rβ/gp130(YFFFFF), pRcCMV-IL-5Rβ/gp130(YF)-SHP2 chimera were described previously [13]. pRcCMV-IL-5Rβ/gp130(YF)/PTP encodes a C-terminal deletion mutant of IL-5Rβ/gp130(YFYYYY) lacking amino acids 766–918 of gp130 with a fused fragment of SHP2 protein (PTP-domain) lacking the N-terminal 208 amino acids [14]. EpoR/gp130 receptor chimeras (pRcCMV-EG (YYYYYY), pRcCMV-EG (YFYYYY) and pRcCMV-EG (FYFFFF)) were described previously [15, 16].

Gab1 wild-type and mutants were derived from pBAT-Gab1 [6] and subcloned into pd2eGFP-Gab1 and pcDNA3. The expression vectors for all Gab1-GFP-fusion proteins were derived from the pd2eGFP-N1 expression vector (Clontech, USA) and were described previously [10]. pd2eGFP-Gab1-WT encodes wild-type Gab1. pd2eGFP-Gab1-Y627/659F encodes a Gab1 mutant where tyrosine 627 and 659 were replaced by phenylalanine to impair binding of SHP2 [9]. pd2eGFP-Gab1-ΔGrb2 codes for a Gab1 mutant which does not bind Grb2. In Gab1-ΔGrb2 the amino acids 341–348 are deleted and leucine 524 was replaced by proline [17]. The coding sequences for Gab1-WT, Gab1-Y627/659F and Gab1-ΔGrb2 were also cloned into pcDNA5/FRT/TO (ThermoFisher, USA). The encoded proteins do not contain C-terminal GFP-tags. All Gab1 constructs used in this study contain an N-terminal Flag-tag.

### Cell culture

Murine embryonal fibroblasts of wild-type mice (MEF-WT) or of Gab1-deficient mice (MEF-Gab1−/−) (kindly provided by W. Birchmeyer [18]) were grown in DMEM, supplemented with FCS (10%), streptomycin (100 mg/l) and penicillin (60 mg/l), at 37 °C in a water-saturated atmosphere in the presence of 5% CO_2_.

HEK293 cells of the FlpIn™ T-REx™ 293 cell line (ThermoFisher Scientific, USA) were grown in Dulbecco’s modified Eagle’s medium (DMEM, Life Technologies, Germany), supplemented with FCS (10%), streptomycin (100 mg/l), penicillin (60 mg/l), zeocin (100 μg/ml), and blasticidin (5 μg/ml) in a water-saturated atmosphere in the presence of 5% CO_2_ at 37 °C.

Primary normal human dermal fibroblasts (NHDF) (kindly provided by Hans F. Merk and Yvonne Marquardt, Medical School RWTH Aachen) were isolated from foreskin as described previously [19, 20]. After isolation, NHDFs were grown in DMEM, supplemented with FCS (10%), streptomycin (100 mg/l) and penicillin (60 mg/l) at 37 °C in a water-saturated atmosphere in the presence of 10% CO_2_.

### Generation of human Gab1 knock-out cells

HEK293 cells of the FlpIn™ T-REx™ 293 cell line (ThermoFisher Scientific, USA) were targeted with CRISPR/Cas9 to induced knock-out of Gab1. Therefore, a Gab1 CRISPR/Cas9 KO plasmid (Santa Cruz, USA) coding for a pool of three different guide RNAs targeting the Gab1 gene and coding for Cas9 was transfected into FlpIn™ T-REx™ 293 cells. A Gab1 homology-directed repair (HDR) plasmid coding for a puromycin resistance flanked by a loxP site was co-transfected. Guide RNA- and Cas9-induced DNA double strand breaks activate the HDR pathway leading to directed integration of the loxP flanked puromycin resistance into the genome and thereby destruction of the genomic Gab1 coding sequence. After picking single clones and subsequent selection of puromycin-resistent cell clones, Gab1 knock-out was confirmed by RT-qPCR and Western Blot. The engineered cell line entitled HEK293 Gab1-KO is grown in DMEM, supplemented with FCS (10%), streptomycin (100 mg/l), penicillin (60 mg/l), zeocin (100 μg/ml), puromycin (5 μg/ml) and blasticidin (5 μg/ml) in a water-saturated atmosphere in the presence of 5% CO_2_ at 37 °C.

### Reconstitution of human Gab1 knock-out cells with Gab1-WT, Gab1-Y627/659F or Gab1-ΔGrb2

Stable HEK293 Gab1-KO cells were engineered for doxycycline-dependent expression of Gab1-WT, Gab1-Y627/659F, or Gab1-ΔGrb2. HEK293 Gab1-KO cells were transfected with pcDNA5/FRT/TO (ThermoFisher Scientific, USA) expression vectors coding for Gab1-WT, Gab1-Y627/659F, or Gab1-ΔGrb2 according to manufacturer’s protocol for stable protein expression in FlpIn™ T-REx™ 293 cell line (ThermoFisher Scientific, USA). Expression of Gab1-WT, Gab1-Y627/659F, or Gab1-ΔGrb2 in selected clones was confirmed by Western Blot. HEK293 Gab1-rec, HEK293 Gab1-Y627/659F-rec, and HEK293 Gab1-ΔGrb2-rec cells were grown in DMEM, supplemented with FCS (10%), streptomycin (100 mg/l), penicillin (60 mg/l), hygromycin B (50 μg/ml) and blasticidin (5 μg/ml) in a water-saturated atmosphere in the presence of 5% CO_2_ at 37 °C.

### Transfection

pd2eGFP-N1 expression vectors for Gab1-WT-GFP and Gab1-ΔGrb2-GFP as well as pRcCMV expression vectors for the IL-5R/gp130 receptor chimeras (IL-5Rα/ gp130(YFYYYY), IL-5Rβ/gp130(YFYYYY), IL-5Rα/gp130(YYFFFF), IL-5Rβ/gp130(YFFFFF), and IL-5Rβ/gp130(YF)-SHP2) or for EpoR/gp130 receptor chimeras (EG (YYYYYY), EG (YFYYYY), and EG (FYFFFF)) were transiently transfected into HEK293, HEK293 Gab1-KO and HEK293 Gab1-rec cells using Lipofectamine 2000 (Life Technologies, Germany) according to manufacturer’s instructions.

### Western blotting

Cells were lysed in RIPA lysis buffer (50 mM Tris-HCl pH 7.4, 150 mM NaCl, 0.5% Nonidet P-40/Igepal, 15% glycerol), supplemented with NaF (1 mM), Na_3_VO_4_ (1 mM), AEBSF (0.8 μM) (Roth, Germany) and aprotinin, pepstatin (SigmaAldrich, USA), leupeptin (MP Biochemicals, Germany) (10 μg/ml of each). Protein concentrations of lysates were determined by Biorad Protein Assay (Biorad, Germany). Equal quantities of protein were separated by SDS-PAGE and transferred to a nitrocellulose membrane. After blocking with Roti®Block (Roth, Germany), membranes were incubated with specific primary antibodies (1:1000) and subsequently incubated with a secondary IRDye 800CW-conjugated anti-rabbit or anti-goat or IRDye 680RD-conjugated anti-mouse antibody (LI-COR, USA). Proteins were visualized using the LI-COR Odyssey Infrared Imaging System (LI-COR, USA).

### Protein immunoprecipitation

Cell lysates [30 μg] were incubated with antibodies (1 μg) specific for the protein of interest at 4 °C over night. Protein G-Dynabeads (Life Technologies, USA) were added for an additional 4 h. Complexes were isolated with a magnet and washed three times with 1 ml RIPA washing solution (50 mM Tris/HCl pH 7.4, 100 mM NaCl, 1 mM EDTA, 0.1% Nonidet P-40/Igepal). Finally, proteins were resolved and analysed by SDS-PAGE and Western blotting.

### Confocal laser-scanning microscopy

HEK293 Gab1-KO cells were seeded on poly-L-lysine-coated glass cover slips and cultivated for 24 h. Subsequently, cells were transfected with expression vectors for Gab1-WT-eGFP and EpoR/gp130 receptor chimeras. On the next day, cells were placed into the pre-heated incubation chamber of the laser scanning microscope and left for 30 min. Where indicated, cells were additionally stimulated with Epo (7 U/ml) as noted in the legend for the specific figures. Imaging was performed with a confocal laser scanning microscope (LSM700, Zeiss, Jena, Germany). The temperature of the incubation chamber (Pecon, Erbach, Germany) and of the microscope’s objective was adjusted to 37 °C; the atmosphere was set to 5% CO_2_. eGFP-fusion proteins were excited using laser light of λ = 488 nm. Emission was detected in the range of λ = 493 to 700 nm.

### Quantitative real-time PCR

Total RNA was isolated using the GeneJET RNA Purification Kit (ThermoFisher Scientific, USA) according to manufacturer’s instructions. RNA (500 ng) was reverse transcribed into cDNA with RevertAid RT Reverse Transcription Kit (ThermoFisher Scientific, USA) using random hexameric primers (ThermoFisher Scientific, USA) according to manufacturer’s instructions. Gene expression of c-Fos, SOCS3 and SDHA was measured with primers for human c-Fos (fw: 5′-GCA TTA CAG AGA GGA GAA ACA C-3′, rev: 5′-AGA AAA GAG ACA CAG ACC CAG-3′), human SOCS3 (fw: 5′-GGA GTT CCT GGA CCA GTA CG-3′, rev: 5′-TTC TTG TGC TTG TGC CAT GT-3′) and human SDHA (fw: 5′- TGG GAA CAA GAG GGC ATC TG - 3′, rev: 5′- CCA CCA CTG CAT CAA ATT CAT G -3′). PCR was performed using Maxima SYBR Green qPCR Master Mix (ThermoFisher Scientific, USA) according to manufacturer’s instructions. Quantification of gene expression was calculated as described by Pfaffl et al. [21].

## Results

### Gab1 enhances and prolongs IL-6-induced MAPK activation

**Fig. 1:**
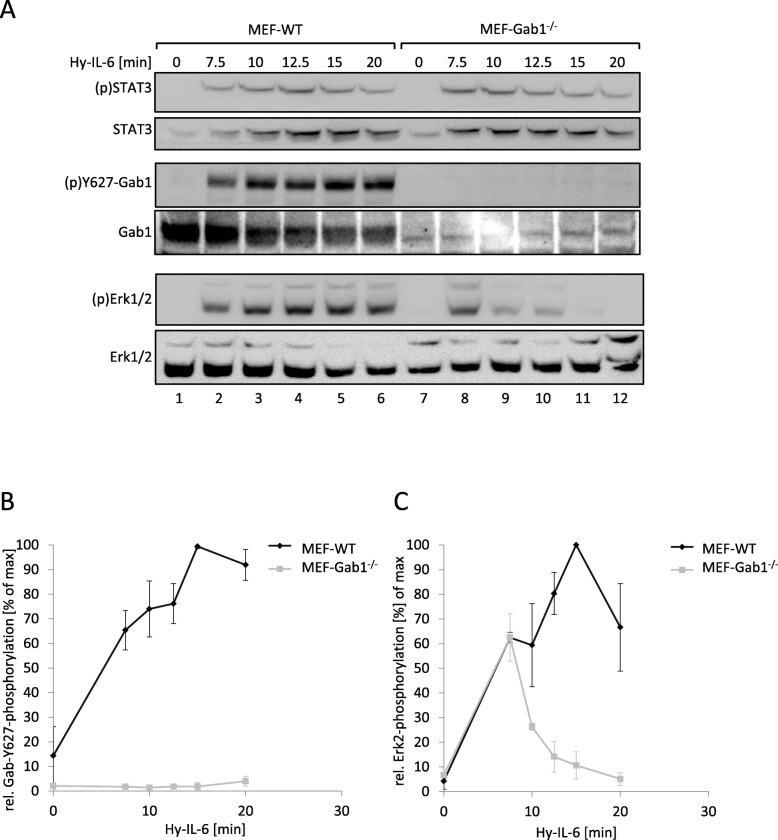
IL-6-induced Erk1/2 pathway activation is amplified by Gab1 in murine embryonic fibroblasts. **a** Murine embryonic fibroblast (MEF-WT) and Gab1-deficient murine embryonic fibroblast (MEF-Gab1^−/−^) cells were seeded and cultivated for 24 h. On the following day, cells were serum-starved for 4 h and subsequently stimulated with Hy-IL-6 (100 ng/ml) for the indicated times. Subsequently, cell lysates were prepared and proteins separated by SDS-PAGE. After Western blotting, membranes were stained for (p)STAT3, STAT3, (p)Y627-Gab1, Gab1, (p)Erk1/2 and Erk1/2. Representative results of *n* = 3 independent experiments are shown. **b** For quantification of Gab1 Y627 phosphorylation signals of (p)Y627-Gab1 and Gab1 were analysed via densitometry. The diagram shows the ratio of (p)Y627-Gab1 to Gab1 for each time point. Maximal phosphorylation of Gab1 Y627 in each experiment was set to 100%. Data are given as mean of three independent experiments ± SD. **c** For quantification of Erk2 phosphorylation, signals of (p)Erk2 and Erk2 were analysed via densitometry. The diagram shows the ratio of (p)Erk2 to Erk2 for each time point. Maximal phosphorylation of Erk2 in each experiment was set to 100%. Data are given as mean of three independent experiments ± SD

Gab1 plays a dual role in IL-6-induced MAPK/Erk pathway activation. On the one hand Gab1 is activated by MAPK-dependent phosphorylation [10], on the other hand Gab1 contributes to MAPK activity [22]. Therefore, we asked whether Gab1 impacts on the temporal dynamics of IL-6-dependent MAPK activity. We compared the kinetics of MAPK activation in murine embryonic fibroblasts (MEF) and corresponding MEF cells from Gab1-deficient mice (MEF-Gab1^−/−^ cells). MEF cells do not express the IL-6Rα and therefore do not respond to IL-6 alone. However, they respond to IL-6 trans-signalling, initiated by a complex of sIL-6Rα and IL-6. The designer fusion protein Hyper-IL-6 (Hy-IL-6) consists of IL-6 which is fused to sIL-6Rα and thus substitutes for the IL-6:sIL-6Rα-complex [23]. Treatment of MEF cells with Hy-IL-6 induces phosphorylation of STAT3 (Y705), Gab1 (Y627) and Erk1/2 (T202,Y204) (Fig. 1a, lanes 1–6), showing that Hy-IL-6 is able to induce IL-6 signalling in cells not expressing IL-6Rα. As expected, Gab1 expression and phosphorylation is not detectable in MEF-Gab1^−/−^ cells (Fig. 1a, quantification of Gab1 tyrosine phosphorylation in Fig. 1b). Hy-IL-6-induced phosphorylation of STAT3 is not altered in MEF-Gab1^−/−^ cells (Fig. 1a, top panel, lanes 7–12), demonstrating that STAT3 activation is independent of Gab1 expression. However, IL-6-induced Erk1/2 phosphorylation is strongly reduced and very transient in Gab1-deficient MEF cells, stimulated for up to 20 min. Of note, the initial Erk1/2 phosphorylation 7.5 min post stimulation is not affected in Gab1 deficient cells (Fig. 1a, see Fig. 1c for quantification of Erk2 phosphorylation).

**Fig. 2:**
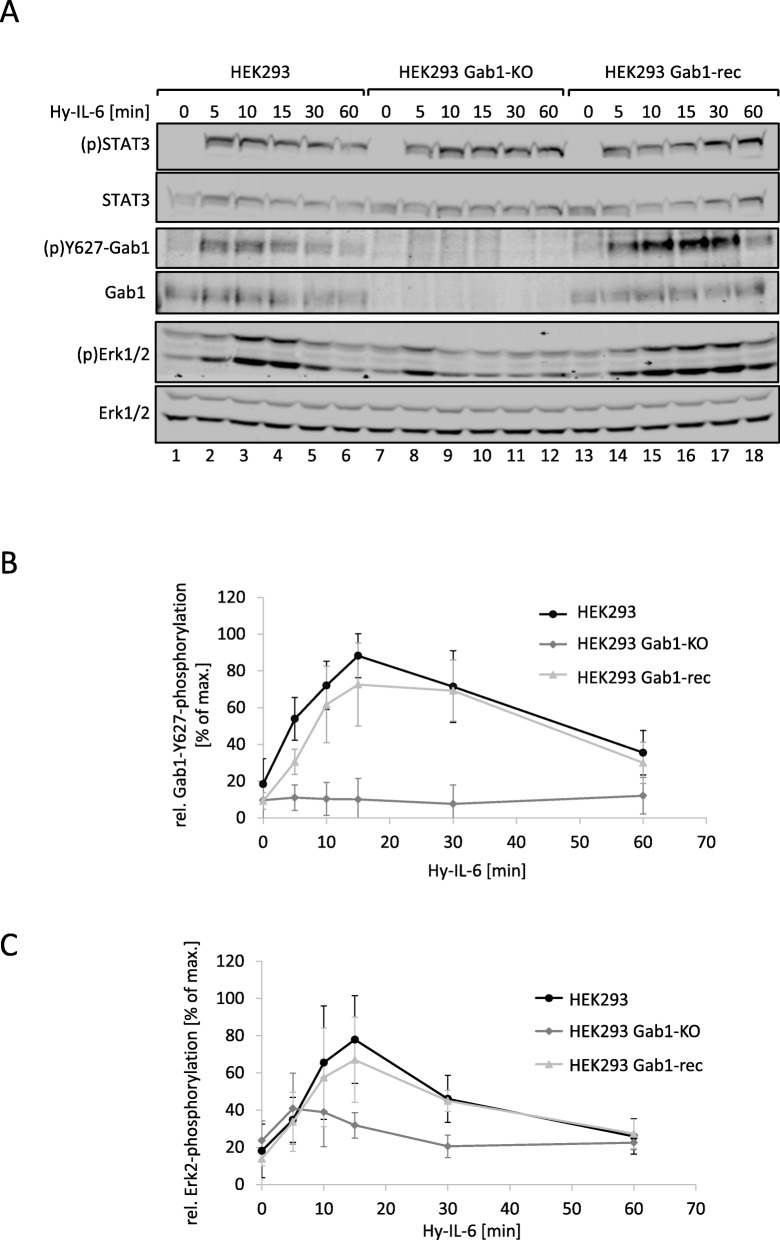
Reconstitution of Gab1 expression in human Gab1-deficient cells rescues IL-6-induced Erk1/2 pathway activation. **a** HEK293, HEK293 Gab1-KO, and HEK293 Gab1-rec cells were seeded and cultivated for 24 h. On the following day, cells were serum-starved for 4 h and subsequently stimulated with Hy-IL-6 (50 ng/ml) for the times, indicated. Subsequently, cell lysates were prepared and proteins separated by SDS-PAGE. After Western blotting, membranes were stained for (p)STAT3, STAT3, (p)Y627-Gab1, Gab1, (p)Erk1/2, and Erk1/2. Representative results of *n* = 3 independent experiments are shown. **b** For quantification of Gab1 Y627 phosphorylation signals of (p)Y627-Gab1 and Gab1 were analysed via densitometry. The diagram shows the ratio of (p)Y627-Gab1 and Gab1 for each time point. Maximal phosphorylation of Gab1 in each experiment was set to 100%. Data are given as mean of three independent experiments ± SD. **c** For quantification of Erk2 phosphorylation signals of (p)Erk2 and Erk2 were analysed via densitometry. The diagram shows the ratio of (p)Erk2 and Erk2 for each time point. Maximal phosphorylation of Erk2 in each experiment was set to 100%. Data are given as mean of three independent experiments ± SD

We also depleted the Gab1 gene in human embryonic kidney (HEK293) cells using a CRISPR/Cas9 approach. The resulting HEK293 Gab1-KO cells were reconstituted to re-express Gab1 (HEK293 Gab1-rec) for control. Gab1 expression levels were comparable in HEK293 cells and HEK293 Gab1-rec cells. We monitored Hy-IL-6-induced phosphorylation of STAT3, Gab1, and Erk1/2 in HEK293 cells, HEK293 Gab1-KO cells, and in HEK293 Gab1-rec cells for up to 60 min (Fig. 2a, see Fig. 2b and c for quantification of Gab1 tyrosine phosphorylation and Erk2 phosphorylation, respectively). As already demonstrated for Gab1-deficient MEF cells in Fig. 1, STAT3 activation is not affected by Gab1 deficiency (Fig. 2a, top panel). Gab1 expression and phosphorylation is not detectable in Gab1-depleted HEK293 cells (Fig. 2a, see Fig. 2b for quantification of Gab1 tyrosine phosphorylation). Again, depletion of Gab1 does not affect early Erk1/2 phosphorylation (Fig. 2a, lane 8). However, in these Gab1-deficient HEK293 cells Erk1/2 activation is dampened 10 min after stimulation and beyond Fig. 2a, lanes 9 to 12). HEK293 Gab1-rec cells show similar activation kinetics to HEK293 cells (for quantification of Erk2 phosphorylation see Fig. 2c).

From these results, we conclude that Gab1 contributes to late and sustained Erk1/2 activation in response to IL-6, whereas it does not contribute to early Erk1/2 activation.

### IL-6-induced SHP2 and Erk1/2 phosphorylation depends on tyrosine 759 within gp130

Based upon the results described above, we assumed that late Erk1/2 activation depends on Gab1, whereas early Erk1/2 activation is receptor-mediated. Recruitment of SHP2 to activated and phosphorylated gp130 is crucial for IL-6-induced MAPK pathway activation [4]. Thus, we investigated whether the SHP2-binding site tyrosine 759 within gp130 contributes to IL-6-induced early or late phosphorylation of Erk1/2 in the presence or absence of Gab1. We transiently expressed chimeric receptors consisting of the extracellular part of the erythropoietin receptor (EpoR) and the transmembrane and cytosolic parts of wild-type or mutated gp130 in HEK293, HEK293 Gab1-KO, and HEK293 Gab1-rec cells. The use of EpoR/gp130 chimeric receptors allowed us to analyse signalling through mutated receptors, independently from ubiquitously expressed, endogenous gp130. Erythropoietin (Epo) as a stimulant to induce IL-6 signal transduction acts only on cells expressing the chimeric receptors but does not activate endogenous gp130. The experimental setup using EpoR/gp130 chimeric receptors has been characterised in considerable detail and is a widely used method [13, 16].

**Fig. 3:**
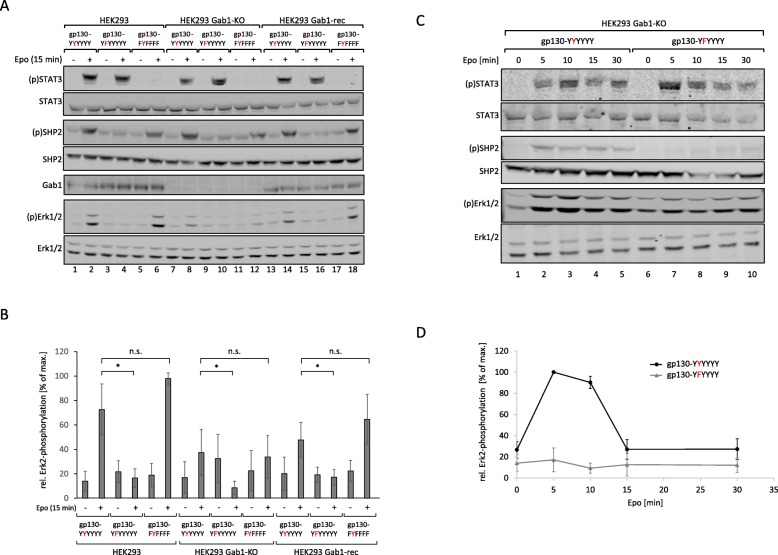
IL-6-induced Erk pathway activation depends on phosphorylation of Y759 in gp130 .**a** HEK293, HEK293 Gab1-KO and HEK293 Gab1-rec cells were seeded and transfected with expression vectors (0.4 μg) for the EpoR/gp130 chimeric proteins EG (YYYYYY), EG (YFYYYY) or EG (FYFFFF). After 24 h, cells were serum-starved for 4 h and were stimulated with Epo (7 U/ml) for 15 min. Subsequently, cell lysates were prepared and proteins separated by SDS-PAGE. After Western blotting, membranes were stained for (p)STAT3, STAT3, (p)SHP2, SHP2, Gab1, (p)Erk1/2, and Erk1/2. Representative results of *n* = 5 independent experiments are shown. **b** For quantification of Erk2 phosphorylation, signals of (p)Erk2 and Erk2 were analysed via densitometry. The diagram shows the ratio of (p)Erk2 and Erk2. Maximal phosphorylation of Erk2 in each experiment was set to 100%. Data are given as mean of five independent experiments ± SD. Student’s t-test: n.s. = non-significant, *p < 0.05. **c** HEK293 Gab1-KO cells were seeded and transfected with expression vectors (0.4 μg) for the EpoR/gp130 chimeric proteins EG (YYYYYY) or EG (YFYYYY). After 24 h, cells were serum-starved for 4 h. Cells were stimulated with Epo (7 units/ml) for the indicated times. Cell lysates were prepared and proteins separated by SDS-PAGE. After Western blotting, membranes were stained for (p)STAT3, STAT3, (p)SHP2, SHP2, (p)Erk1/2, and Erk1/2. Representative results of *n* = 3 independent experiments are shown. **d** For quantification of Erk2 phosphorylation, signals of (p)Erk2 and Erk2 were analysed via densitometry. The diagram shows the ratio of (p)Erk2 and Erk2 for each time point. Maximal phosphorylation of Erk2 in each experiment was set to 100%. Data are given as mean of three independent experiments ± SD

As expected, stimulation of HEK293 cells, which express EpoR/gp130(YYYYYY) chimeric wild-type receptors, induces phosphorylation of STAT3, SHP2 (Y542), and Erk1/2 (Fig. 3a, lanes 1–2). Substitution of the SHP2 recruitment site tyrosine 759 within gp130 by phenylalanine (EpoR/gp130(YFYYYY)) eliminates ligand-induced SHP2 and Erk1/2 phosphorylation. STAT3 phosphorylation is not affected (Fig. 3a, lanes 3–4). Of note, activation of chimeric receptors lacking all cytoplasmic tyrosine motifs but containing tyrosine 759 (EpoR/gp130(FYFFFF)), does not initiate STAT3 phosphorylation but retains SHP2 and Erk1/2 phosphorylation (Fig. 3a, lanes 5–6). These data confirm that the tyrosine 759 motif within gp130 is required and sufficient to translate ligand-induced receptor activation into SHP2 phosphorylation and Erk1/2 activation.

To evaluate the contribution of Gab1 to SHP2 and Erk1/2 phosphorylation, we monitored ligand-dependent signalling through the three chimeric receptors in HEK293 Gab1-KO cells after 15 min of stimulation (Fig. 3a, lanes 7–12). As expected, Gab1 expression is not detectable in these cells. STAT3 and SHP2 phosphorylation are not altered in their response due to the lack of Gab1 expression (Fig. 3a, lane 8). However, Erk1/2 phosphorylation is reduced, although not completely abolished (compare Fig. 3a, lanes 8 and 10; see Fig. 3b for quantification of Erk2 phosphorylation). Re-expression of Gab1 in Gab1 KO cells (HEK293 Gab1-rec cells) reconstitutes the signals detected in HEK293 cells almost completely (Fig. 3a lanes 13 to 18; see Fig. 3b for quantification of Erk2 phosphorylation).

These results confirm that phosphorylation of STAT3 and SHP2 depend on specific, distinct tyrosine motifs within gp130. Phosphorylation of both proteins occurs independently of Gab1. Erk1/2 phosphorylation depends, like SHP2 phosphorylation, on tyrosine 759 within gp130 but it is notably affected by depletion of Gab1. However, a residual Gab1-independent Erk1/2 phosphorylation is also clearly evident.

### Gab1-independent early Erk1/2 activation is transient and depends on tyrosine 759 within gp130

To study Gab1-independent Erk1/2 activation, we first performed kinetic analyses of Erk1/2 phosphorylation in HEK293 Gab1-KO cells, expressing either EpoR/gp130(YYYYYY) or EpoR/gp130(YFYYYY) chimeric receptors. Erk1/2 phosphorylation induced by activated EpoR/gp130-YYYYYY receptors reaches a maximum between 5 and 10 min after stimulation. Substitution of tyrosine 759 by phenylalanine leads to complete loss of ligand-dependent SHP2 and Erk1/2 phosphorylation (Fig. 3c, see Fig. 3d for quantification of Erk2 phosphorylation). These results show that Gab1-independent Erk1/2 phosphorylation is transient as it is detectable only within the first 15 min after stimulation. This early Gab1-independent activation of Erk1/2 crucially depends on tyrosine 759 within gp130.

### SHP2 contributes to early Erk1/2 activation

**Fig. 4:**
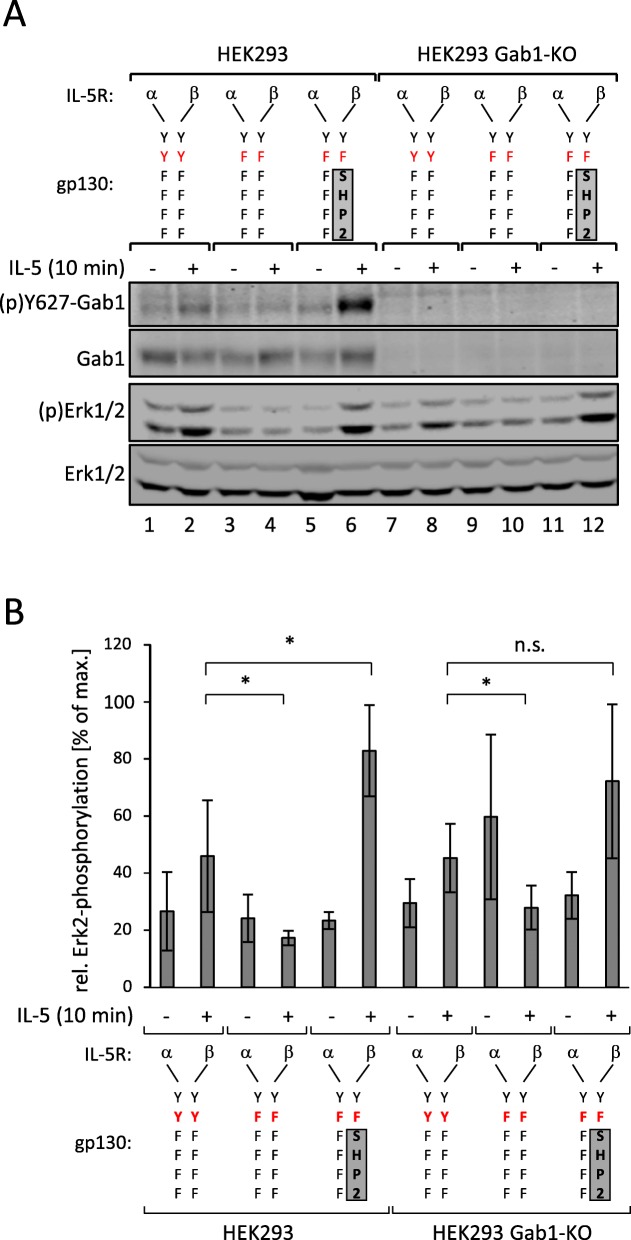
SHP2 binding to gp130 is required for IL-6-induced phosphorylation of Gab1. **a** HEK293 and HEK293 Gab1-KO cells were seeded and transfected with expression vectors (0.4 μg) for IL-5Rα/gp130(YYFFFF) and IL-5Rβ/gp130(YYFFFF), for IL-5Rα/gp130(YFFFFF) and IL-5Rβ/gp130(YFFFFF), or for IL-5Rα/gp130(YFFFFF) and IL-5Rβ/gp130(YF)-SHP2 chimeric receptors. After 24 h, cells were serum-starved for 4 h. Cells were treated with IL-5 (100 ng/ml) for 10 min. Subsequently, cell lysates were prepared and proteins separated by SDS-PAGE. After Western blotting, membranes were stained for (p)Y627-Gab1, Gab1, (p)Erk1/2, and Erk1/2. Representative results of n = 5 independent experiments are shown. **b** For quantification of Erk2 phosphorylation, signals of (p)Erk2 and Erk2 were analysed via densitometry. The diagram shows the ratio of (p)Erk2 and Erk2. Maximal phosphorylation of Erk2 in each experiment was set to 100%. Data are given as mean of five independent experiments ± SD. Student’s t-test: n.s. = non-significant, *p < 0.05

The protein tyrosine phosphatase SHP2 binds to the phosphotyrosine 759-motif within gp130 [4]. The importance of the tyrosine 759-motif for early Erk1/2 phosphorylation suggests that SHP2 is contributing to early Erk1/2 phosphorylation. To test the impact of SHP2 on early Erk1/2 activation we either eliminated SHP2 from the receptor complex by mutating tyrosine 759, or we forced SHP2 into the receptor complex by fusing SHP2 to the mutated receptor (Fig. 4a). We compared Erk1/2 phosphorylation after activation of heterodimeric IL-5R/gp130 chimeric receptor complexes. These receptors either miss all functional tyrosine motifs but retaining tyrosine 759 (IL-5Rα/gp130(YYFFFF): IL-5Rβ/gp130(YYFFFF)) or miss all functional tyrosine motifs (IL-5Rα/gp130(YFFFFF):IL-5Rβ/gp130(YFFFFF)). For forced reconstitution of the SHP2:receptor interaction, the IL-5Rβ receptor is fused to an SHP2 fragment which lacks the N-terminal SH2 domain tandem, but retains the catalytic domain and the entire C-terminal region of SHP2 (IL-5Rα/gp130(YFFFFF):IL-5Rβ/gp130(YF)-SHP2) [14]. Again, we benefit from the use of chimeric receptors, which allow us to activate specifically transfected receptor mutants without activating endogenous gp130.

In line with the results obtained utilising EpoR/gp130 chimera (Fig. 3), the receptor complex containing tyrosine 759 (IL-5Rα/gp130(YYFFFF):IL-5Rβ/gp130(YYFFFF)) induces Erk1/2 phosphorylation in response to IL-5. (Fig. 4a, lane 2; Fig. 4b for quantification of Erk2-phosphorylation). This phosphorylation clearly depends on tyrosine 759, as activation of a receptor complex, which lacks tyrosine 759 (IL-5Rα/gp130(YFFFFF):IL-5Rβ/gp130(YFFFFF)) does not activate Erk1/2 phosphorylation (Fig. 4a, lane 4). However, Erk1/2 phosphorylation can be restored and enhanced by fusing SHP2 to the receptor complex (IL-5Rα/gp130(YFFFFF):IL-5Rβ/gp130(YF)-SHP2) (Fig. 4a, see lane 6; Fig. 4b for quantification of Erk2 phosphorylation). These observations suggest that SHP2 contributes to Erk1/2 activation by binding to phosphorylated tyrosine 759 within the cytoplasmic part of gp130. As shown before, an early, Gab1-independent Erk1/2 phosphorylation is also detectable in HEK293 Gab1-KO cells. This early activation does not depend on Gab1 expression (Fig. 4a, lanes 8 and 12) but requires tyrosine 759 of gp130 and recruitment of SHP2 to gp130.

These results are consistent with a two phase mechanism of Erk1/2 activation in response to IL-6 signalling. Initial Erk1/2 activation is mediated via binding of SHP2 to gp130 and is Gab1-independent. Late phase Erk1/2 activity and amplification of the signal depends on Gab1 expression.

### Requirements for initiation of Gab1-dependent signalling

As we conclude from our prior results that Gab1 contributes to late Erk1/2 activation we asked how Gab1 extends IL-6-induced Erk1/2 pathway activation. Previous own studies already showed that recruitment of Gab1 to PIP3 at the plasma membrane is an essential step for IL-6-induced Erk1/2 activation. Membrane recruitment of Gab1 is regulated by a switch-like mechanism, which depends on Erk1/2-dependent phosphorylation of serine 552 in Gab1. This phosphorylation initiates subsequent conformational changes of Gab1 which relieve the PH domain and thus enable binding of Gab1 to PIP3 at the plasma membrane [9, 10].

**Fig. 5:**
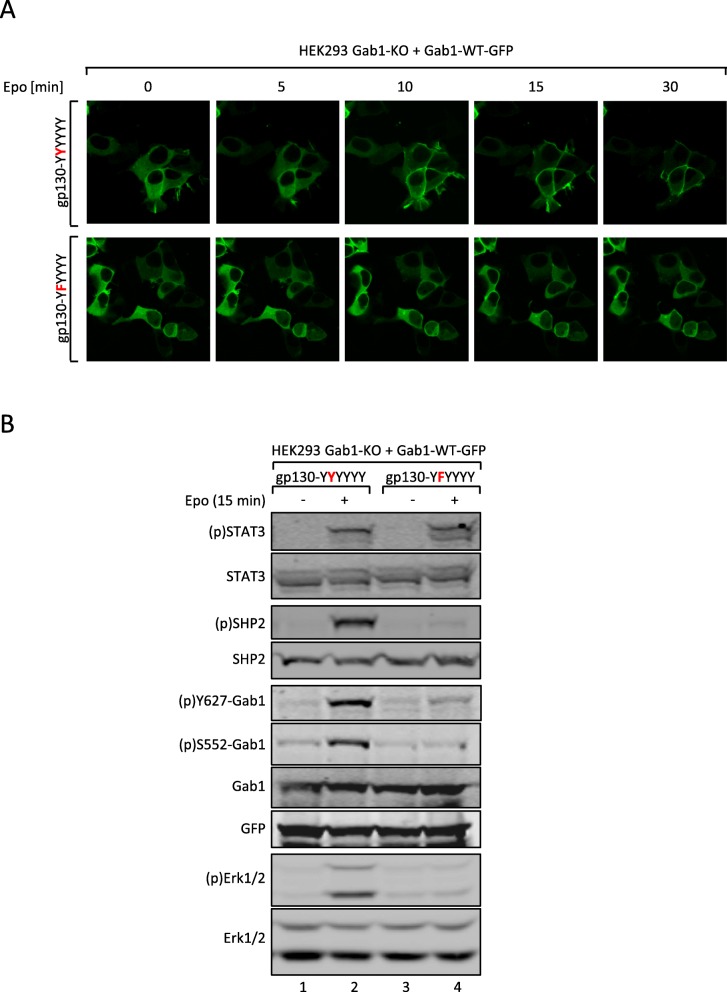
Tyrosine 759 in gp130 is crucial for IL-6-induced Gab1 translocation and phosphorylation **(a)** HEK293 Gab1-KO cells were seeded on poly-L-lysine-coated glass cover slips and co-transfected with expression vectors for Gab1-WT-GFP and either EG (YYYYYY) or EG (YFYYYY). On the following day, cells were serum-starved for 4 h, subsequently placed into the incubation chamber of a laser scanning microscope and left for 30 min. Cells were treated with Epo (7 U/ml) to induce IL-6 signalling. Imaging was performed before and after treatment with Epo for up to 30 min as indicated. **b** HEK293 Gab1-KO cells were seeded and co-transfected with expression vectors for Gab1-WT-GFP and either EG (YYYYYY) or EG (YFYYYY). After 24 h, cells were serum-starved for 4 h. Cells were left untreated or treated with Epo (7 U/ml) for 15 min. Subsequently, cell lysates were prepared and proteins separated by SDS-PAGE. After Western blotting, membranes were stained for (p)STAT3, STAT3, (p)SHP2, SHP2, (p)Y627-Gab1, (p)S552-Gab1, Gab1, (p)Erk1/2, and Erk1/2. After stripping, membranes were stained for GFP. Representative results of *n* = 3 independent experiments are shown

We tested whether the initial, gp130 tyrosine 759-dependent Erk1/2 pathway activation is necessary for the recruitment of Gab1 to the plasma membrane. Therefore, HEK293 Gab1-KO cells were transiently transfected with expression vectors for Gab1-WT-GFP and for either EpoR/gp130(YYYYYY) or EpoR/gp130(YFYYYY) chimeric receptors. Membrane localization of Gab1-WT-GFP was monitored by confocal laser-scanning microscopy of living cells (Fig. 5a). Activation of EpoR/gp130(YYYYYY) initiates membrane recruitment of Gab1-WT-GFP within 15 min post stimulation. In contrast, activation of EpoR/gp130(YFYYYY) does not affect the cytoplasmic distribution of Gab1-GFP. These observations in Gab1-deficient cells reconstituted with Gab1-GFP-fusion proteins are in line with our own previous studies, made in cells expressing Gab1-GFP in addition to the endogenous Gab1 protein [9]. These results clearly show the need of gp130-tyrosine 759-dependent signalling for triggering Gab1-binding to the plasma membrane.

The impact of tyrosine 759 within gp130 on the phosphorylation of Gab1 at serine 552 and tyrosine 627 was additionally tested by Western blotting with phosphosite-specific antibodies (Fig. 5b). Activation of the EpoR/gp130(YYYYYY) chimeric receptors induces phosphorylation of STAT3, SHP2, Gab1-Y627, Gab1-S552, and Erk1/2. However, phosphorylation of SHP2, Gab1-Y627, and Gab1- S552, and Erk1/2 are lost by mutating Y759 within the receptor. These results show the importance of the SHP2 binding site tyrosine 759 in gp130 to initiate Gab1-dependent signalling. Gab1 signalling is enabled by Gab1 serine 552 phosphorylation, subsequent translocation to the plasma membrane, and tyrosine phosphorylation.

### Impact of Gab1 and Gab1 tyrosine-627 phosphorylation on late IL-6-dependent Erk1/2 pathway activation

**Fig. 6:**
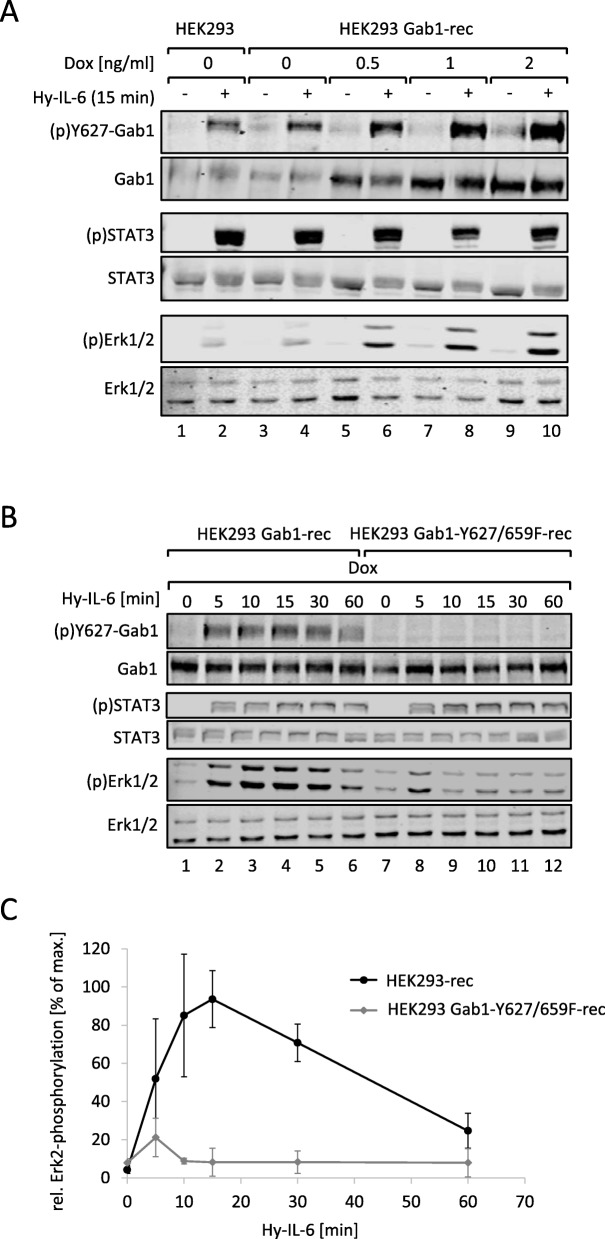
IL-6-induced phosphorylation of Y627 in Gab1 is crucial for Gab1-dependent Erk1/2 pathway amplification. **a** HEK293 and HEK293 Gab1-rec cells were seeded and cultivated for 24 h. On the following day, cells were serum starved. HEK293 cells were left untreated whereas HEK293 Gab1-WT cells were treated with increasing amount of doxycycline (Dox) as indicated in the figure for 4 h to induce dose-dependently Gab1-WT expression. Subsequently, cells were stimulated with Hy-IL-6 (50 ng/ml) for 15 min. Subsequently, cell lysates were prepared and proteins separated by SDS-PAGE. After Western blotting, membranes were stained for (p)Y627-Gab1, Gab1, (p)STAT3, STAT3, (p)Erk1/2 and Erk1/2. Representative results of *n* = 3 independent experiments are shown. **b** HEK293 Gab1-rec and HEK293 Gab1-Y627/659F-rec cells were seeded and cultivated for 24 h. On the following day, all cells were serum starved and were additionally treated with doxycycline (0.5 ng/ml) for 4 h. Subsequently, cells were stimulated with Hy-IL-6 (50 ng/ml) for the times, indicated. Cell lysates were prepared and proteins separated by SDS-PAGE. After Western blotting, membranes were stained for (p)Y627-Gab1, Gab1, (p)STAT3, STAT3, (p)Erk1/2, and Erk1/2. Representative results of *n* = 3 independent experiments are shown. **c** For quantification of Erk2 phosphorylation signals of (p)Erk2 and Erk2 were analysed via densitometry. The diagram shows the ratio of (p)Erk2 and Erk2 for each time point. Maximal phosphorylation of Erk2 was set to 100%. Data are given as mean of three independent experiments ± SD

From the previous results we concluded that the initial IL-6 signalling is crucial for later, Gab1-dependent Erk1/2 activation. To evaluate the impact of Gab1 on Erk1/2 activation in more detail, we studied IL-6-induced Erk1/2 activation in the presence of increasing amounts of Gab1. Gab1 expression in HEK293 Gab1-rec cells increases with increasing amounts of doxycycline. The expression system is leaky in the absence of doxycycline and results in Gab1 expression levels, comparable to those observed for endogenous expression in HEK293 cells (Fig. 6a, compare 2nd panel, lanes 1, 2 with 3, 4). Stimulation of the cells with Hy-IL-6 induces Gab1 tyrosine 627 phosphorylation. The strength of Gab1 phosphorylation correlates with the respective Gab1 expression. Of note, the strength of Erk1/2 phosphorylation also correlates with Gab1 expression and Gab1 tyrosine phosphorylation. These data agree with the hypothesis that Gab1 expression and phosphorylation impacts on Erk1/2 activation in a dose-dependent, rheostat-like manner.

To further evaluate this concept we compared the kinetics of Erk1/2 activation in HEK293 Gab1-rec and HEK293 Gab1-Y627/659F-rec cells. These cells express either Gab1-WT or a Gab1 mutant, which does not bind SHP2 (Gab1-Y627/659F). In cells reconstituted to express Gab1-WT, Hy-IL-6 initiates phosphorylation of STAT3, Gab1-Y627 and Erk1/2 after 5 min of stimulation. Whereas STAT3 phosphorylation is sustained, Gab1 tyrosine 627 phosphorylation and Erk1/2 phosphorylation decline 60 min post stimulation (Fig. 6b). As expected, cells reconstituted with Gab1-Y627/659F exhibited no phosphorylation of Gab1 tyrosine 627. Whereas STAT3 phosphorylation was not affected by the mutation within Gab1, Erk1/2 phosphorylation was much weaker and considerably more transient in cells expressing Gab1-Y627/659F than in cells expressing Gab1-WT (Fig. 6c for quantification of Erk2 phosphorylation). These results show the crucial function of the phosphorylated SHP2-binding site tyrosine 627 within Gab1 for enhanced and sustained Erk1/2 activation.

### Gab1 interaction with Grb2 is required for Gab1 tyrosine 627 phosphorylation and Gab1-dependent Erk1/2 pathway activation

**Fig. 7:**
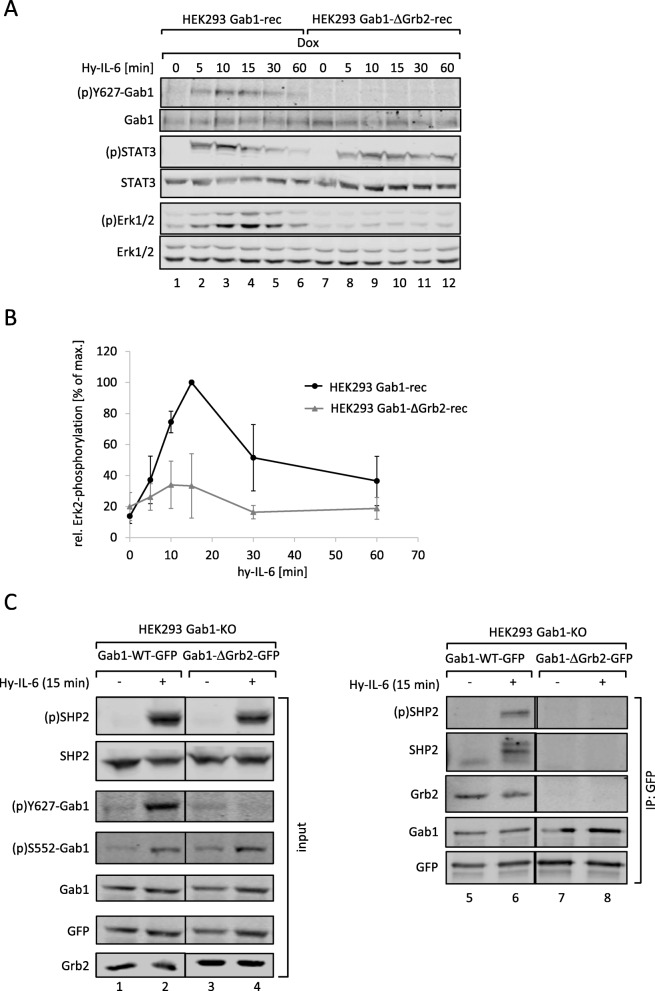
Loss of constitutive interaction of Gab1 and Grb2 impairs IL-6-induced phosphorylation of Y627 in Gab1 and Erk1/2 pathway activation. **a** HEK293 Gab1-rec and HEK293 Gab1-ΔGrb2-rec cells were seeded and cultivated for 24 h. On the following day, all cells were serum starved and additionally treated with doxycycline (0.5 ng/ml) for 4 h to induce expression of Gab1-WT or Gab1-ΔGrb2, respectively. Subsequently, cells were stimulated with Hy-IL-6 (50 ng/ml) for the times, indicated. Cell lysates were prepared and proteins separated by SDS-PAGE. After Western blotting, membranes were stained for (p)Y627-Gab1, Gab1, (p)STAT3, STAT3, (p)Erk1/2, and Erk1/2. Representative results of *n* = 3 independent experiments are shown. **b** For quantification of Erk2 phosphorylation signals of (p)Erk2 and Erk2 were analysed via densitometry. The diagram shows the ratio of (p)Erk2 and Erk2 for each time point. Maximal phosphorylation of Erk2 was set to 100%. Data are given as mean of three independent experiments ± SD. **c** HEK293 Gab1-KO cells were seeded and transfected with expression vectors (0.4 μg) for Gab1-WT-GFP or Gab1-ΔGrb2-GFP. 24 h after transfection, cells were serum-starved for 4 h. Cells were stimulated with Hy-IL-6 (50 ng/ml) for 15 min. Subsequently, cell lysates were prepared to be used for co-immunoprecipitation assays. As input controls (left panels) proteins were separated by SDS-PAGE. After Western blotting, membranes were stained for (p)SHP2, SHP2, (p)Y627-Gab1, (p)S552-Gab1, Gab1, and Grb2. After stripping, membranes were stained for GFP. For co-immunoprecipitation assays (right panels) the proteins were incubated with antibodies against the GFP-tag in Gab1-GFP. Immune complexes were isolated and proteins separated by SDS-PAGE. After Western blotting, membranes were stained for (p)SHP2, Grb2, and Gab1. After stripping, membranes were stained for SHP2 and GFP. Representative results of *n* = 3 independent experiments are shown

Besides SHP2, Grb2 is another potent Erk1/2 pathway component interacting with Gab1. Thus, we investigated the importance of Gab1:Grb2 interaction on late Erk1/2 activation in IL-6-induced signal transduction. We reconstituted HEK293 Gab1-KO cells to express a Gab1 mutant which does not bind Grb2. The resulting HEK293 Gab1-ΔGrb2-rec cells were stimulated for up to 60 min with Hy-IL-6 (Fig. 7a). STAT3 phosphorylation, Gab1-Y627 phosphorylation, and Erk1/2 phosphorylation in these cells were compared to that in Hek293 Gab1-rec cells. Interestingly, a loss of Grb2 binding impaired Gab1 phosphorylation at tyrosine 627 and Erk1/2 phosphorylation (see Fig. 7b for quantification of Erk2 phosphorylation). Thus, mutation of the Grb2 binding site within Gab1 showed similar effects on Erk activation as the mutation of the SHP2 binding site in Gab1-Y627/659F.

The latter observation encouraged us to test whether the mutation of the Grb2 binding site within Gab1 also impacts on the association of Gab1 with SHP2. We performed co-immunoprecipitation assays with GFP-tagged Gab1 proteins as the GFP-tag allows precipitation of Gab1 protein complexes without interfering in the complex formation by Gab1 antibodies. For this approach, HEK293 Gab1-KO cells were reconstituted to express Gab1-WT-GFP or Gab1-ΔGrb2-GFP. The input control confirmed impaired tyrosine 627-phosphorylation in cells expressing Gab1-ΔGrb2 but showed retained serine 552 phosphorylation of Gab1. SHP2 phosphorylation was not affected by the mutation in Gab1 (Fig. 7c, left panels). Gab1-WT-GFP co-precipitated with Grb2 and phosphorylated SHP2. In contrast, Gab1-ΔGrb2-GFP co-precipitated neither with Grb2 nor with SHP2. These observations show that the Gab1:Grb2 interaction contributes to Gab1 tyrosine-627 phosphorylation, SHP2 binding, and Erk1/2 pathway activation.

### Gab1 signalling impacts IL-6-induced MAPK-dependent gene expression in a time-dependent manner

**Fig. 8:**
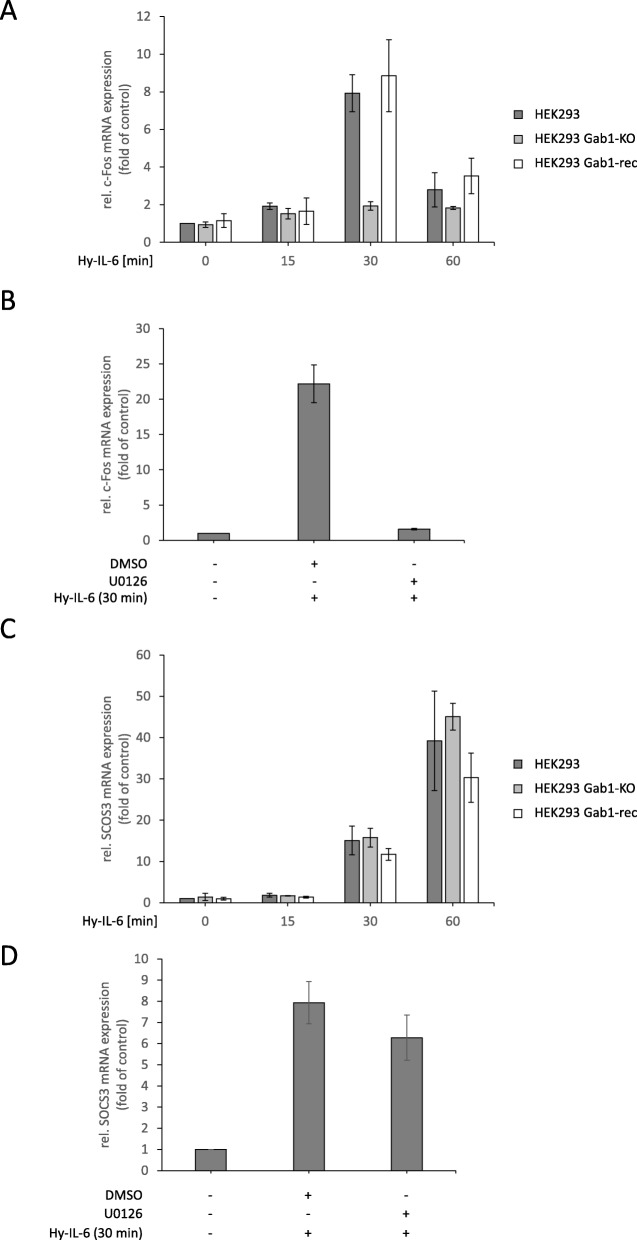
Gab1 signalling impacts IL-6-induced MAPK-dependent gene expression in a time-dependent manner. **a** HEK293, HEK293 Gab1-KO and HEK293 Gab1-rec cells were seeded and cultivated for 24 h. On the following day, cells were serum starved for 4 h and subsequently stimulated with Hy-IL-6 (50 ng/ml) for the times, indicated. Total mRNA was extracted and subjected to qRT-PCR analysis to monitor c-Fos mRNA expression. The expression of c-Fos mRNA was normalized to SDHA mRNA expression. Expression of mRNA is given in fold of mRNA expression in control (=untreated HEK293 cells, set as 1). Data are given as mean of three independent experiments ± SD. **b** Additionally, HEK293 cells were pre-treated with U0126 (10 μM) or its solvent control DMSO for 30 min prior to stimulation with Hy-IL-6 (50 ng/ml, 30 min). Total mRNA was extracted and subjected to qRT-PCR analysis to monitor c-Fos mRNA expression as described for (**a**). (**c, d**) SOCS3 mRNA expression was analysed as described for c-Fos mRNA in (**a**) and (**b**)

To elaborate the impact of Gab1-signalling on IL-6-induced gene expression, we studied the time-dependent induction of c-Fos and SOCS3 mRNA expression in Hy-IL-6-stimulated HEK293 cells, HEK293 Gab1-KO cells, and HEK293 Gab1-rec cells (Fig. 8). Lack of Gab1 expression in HEK293 Gab1-KO cells does not affect early IL-6-induced c-Fos mRNA expression (15 min after stimulation). However, in Gab1-deficient cells, c-Fos mRNA expression is reduced 30 min after stimulation and beyond (Fig. 8a). Inhibition of MEK activity with U0126 demonstrates that IL-6-induced c-Fos mRNA expression at 30 min indeed requires Erk activation. In contrast, SOCS3 mRNA expression is not affected by the loss of Gab1 (Fig. 8c) or MEK inhibition in HEK293 cells (Fig. 8d). These data indicate a gene-specific impact of Gab1-dependent sustained Erk-activation in HEK293 cells.

**Fig. 9:**
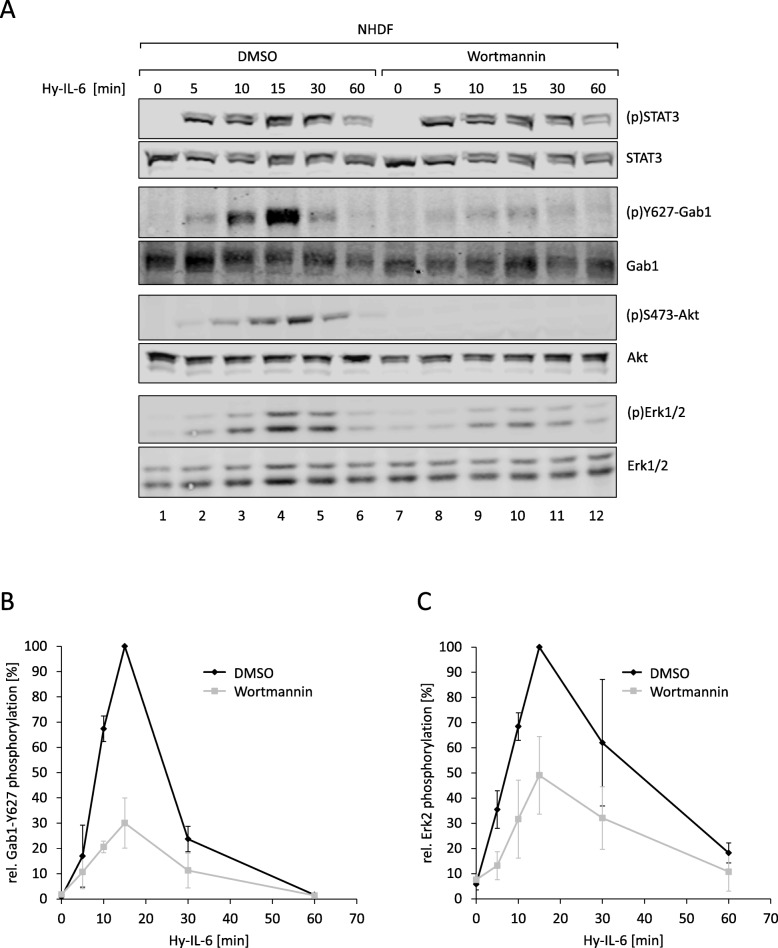
PI3K signalling impacts IL-6-induced Erk activation in normal human dermal fibroblasts in a time-dependent manner. **a** Normal human dermal fibroblasts (NHDF) were seeded and cultivated for 24 h. On the following day, cells were serum starved for 24 h. After serum starvation, NHDF were pre-treated with Wortmannin (100 nM) or its solvent control DMSO for 30 min. Subsequently, cells were stimulated with Hy-IL-6 (50 ng/ml) for the times, indicated. Cell lysates were prepared and proteins were separated by SDS-PAGE. After Western blotting, membranes were stained for (p)STAT3, STAT3, (p)Y627-Gab1, Gab1, (p)S473-Akt, Akt, (p)Erk1/2, and Erk1/2. Representative results of *n* = 3 independent experiments are shown. **b** For quantification of Gab1 Y627 phosphorylation signals of (p)Y627-Gab1 and Gab1 were analysed via densitometry. The diagram shows the ratio of (p)Y627-Gab1 to Gab1 for each time point. Maximal phosphorylation of Gab1 Y627 in each experiment was set to 100%. Data are given as mean of three independent experiments ± SD. **c** For quantification of Erk2 phosphorylation, signals of (p)Erk2 and Erk2 were analysed via densitometry. The diagram shows the ratio of (p)Erk2 to Erk2 for each time point. Maximal phosphorylation of Erk2 in each experiment was set to 100%. Data are given as mean of three independent experiments ± SD

**Fig. 10:**
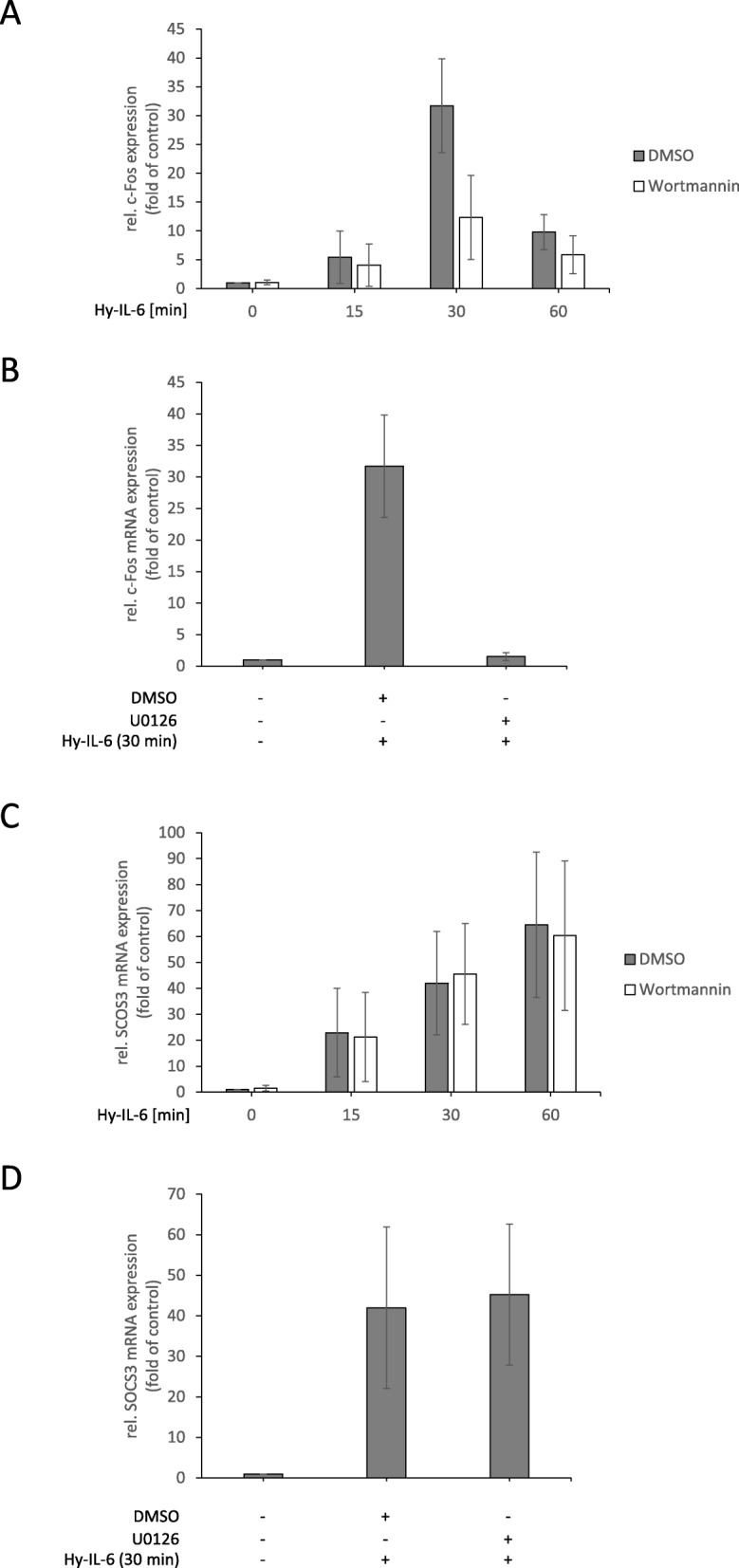
Gab1 signalling impacts IL-6-induced MAPK-dependent gene expression in normal human dermal fibroblasts in a time-in dependent manner. **a** Normal human dermal fibroblasts (NHDF) were seeded and cultivated for 24 h. On the following day, cells were serum starved for 24 h. After serum starvation, NHDF were pre-treated with Wortmannin (100 nM) or its solvent control DMSO for 30 min. Subsequently, cells were stimulated with Hy-IL-6 (50 ng/ml) for the times, indicated. Total mRNA was extracted and subjected to qRT-PCR analysis to monitor c-Fos mRNA expression. The expression of c-Fos mRNA was normalized to SDHA mRNA expression. Expression of mRNA is given in fold of mRNA expression in control (=untreated NHDF, set as 1). Data are given as mean of three independent experiments ± SD. **b** Additionally, NHDF were pre-treated with U0126 (10 μM) or its solvent control DMSO for 30 min prior to stimulation with Hy-IL-6 (50 ng/ml, 30 min). Total mRNA was extracted and subjected to qRT-PCR analysis to monitor c-Fos mRNA expression as described for (**a**). (**c, d**) SOCS3 mRNA expression was analysed as described for c-Fos mRNA in (**a**) and (**b**)

Finally, we analysed time- and Gab1-dependent signalling in normal human dermal fibroblasts (NHDF) derived from primary foreskin dermal fibroblasts. Similar as shown for HEK293 cells, Hy-IL-6 induces the phosphorylation of STAT3, Gab1 and Erk1/2 in NHDF (Fig. 9a, see Fig. 9b, c for quantification of Gab1 and Erk2 phosphorylation). To interfere in Gab1 signalling we added the PI3K inhibitor Wortmannin as we know that Wortmannin blocks membrane localization of Gab1 [9, 10]. Wortmannin efficiently blocks phosphorylation of the PI3K-dependent PDK1-substrate Akt and indeed reduces IL-6-induced Erk1/2 phosphorylation and Gab1 tyrosine phosphorylation most obvious at late time points after 10 min and beyond (Fig. 9a, see Fig. 9b and c for quantification of Gab1 tyrosine phosphorylation and Erk2 phosphorylation, respectively). PI3K inhibition does not affect early c-Fos mRNA expression (15 min after stimulation). However, reduced Gab1 phosphorylation and Erk activation in Wortmannin-treated NHDF cells at later time points correlate with reduced IL-6-induced c-Fos mRNA expression after 30 minas observed in Gab1-deficient HEK293 cells (compare Fig. 10a and Fig. 8a). Figure 10b demonstrates that induction of c-Fos mRNA expression in NHDF cells at 30 min requires Erk activity. In contrast, SOCS3 mRNA expression is unaffected by Wortmannin (Fig. 10c) and does not depend on Erk activation in NHDF cells (Fig. 10d). These results indicate a gene-specific impact of Gab-1-dependent sustained Erk-activation also in normal human dermal fibroblasts.

**Fig. 11:**
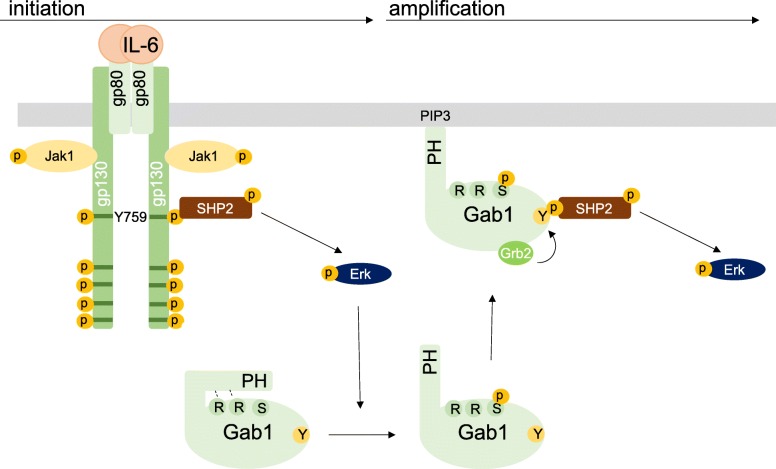
Initiation- and amplification-phase model of IL-6-induced Erk1/2 pathway activation. Initial ligand-induced activation of gp130 leads to phosphorylation of tyrosine residues in gp130, enabling recruitment of SHP2 to phosphorylated tyrosine 759 to activate the Erk1/2 pathway in the initiation phase. Activated Erk phosphorylates serine 552 within Gab1. This phosphorylation releases the intramolecular block of the PH domain and enables recruitment of Gab1 to PIP3 at the plasma membrane. Gab1 recruited at the plasma membrane becomes tyrosine phosphorylated. Phosphorylated tyrosine 627 and 659 within Gab1 serve as binding sites for SHP2. Activation of Erk1/2 in the Gab1-dependent amplification phase requires binding of SHP2 and Grb2 to Gab1. Furthermore, binding of SHP2 to Gab1 depends on binding of Grb2 to Gab1

All these data corroborate our hypothesis of a gp130 tyrosine 759/SHP2-dependent and Gab1-independent initial phase of Erk1/2 activation and a subsequent Gab1-dependent amplification phase of Erk1/2 activation in IL-6 signalling (Fig. 11).

## Discussion

The multi-site docking protein Gab1 orchestrates the PI3K-, MAPK-, PLC-, and Rho family GTPase signalling. Gab1 is a key regulator of growth factor-induced pathway activation and coordinates e.g. EGF- [7, 24] and HGF-induced [17, 24] Erk1/2 activation (for a review see [5]). However, Gab1 also regulates Erk1/2 activation induced by cytokines such as IL-6 [22] and Epo [25].

Our own studies demonstrated constitutive, malregulated Gab1 activation in JAK2-V617F-positive erythroleukaemia cells [11] and reduced proliferation of these cells in the absence of Gab1 [12]. The deletion of Gab1 protein elicits multiple developmental defects. Itoh et al. investigated the role of Gab1 expression in murine embryogenesis and found that Gab1 expression is essential for correct prenatal heart and skin development in murine embryos. Furthermore, loss of Gab1 expression is associated with a reduction of IL-6-induced Erk1/2 phosphorylation in murine fibroblasts [26]. Therefore, it is not surprising that loss of Gab1 expression is lethal [26]. These observations urged us to analyse the molecular mechanisms of IL-6-induced Gab1 activation and its function in regulating Erk1/2 activation and MAPK-dependent gene expression in more detail.

Loss of Gab1 expression in murine embryonic fibroblasts as well as in human embryonal kidney cells is correlated with a reduction of late IL-6-induced Erk1/2 phosphorylation (Figs. 1A, 2A). Notably, Erk1/2 phosphorylation is not affected by Gab1 depletion at the initial phase of signalling but becomes evident later (Figs. 1C, 2C). Thus, we conclude that Gab1 does not impact on the initial Erk1/2 activation, but rather ensures sustained, amplified Erk1/2 activation. We hypothesise a Gab1-independent initial phase of Erk1/2 activation and a Gab1-dependent amplification phase of Erk1/2 activation in response to IL-6 signalling (Fig. 8). To substantiate these ideas we compared the requirements for both phases of Erk1/2 phosphorylation in wild-type cells, Gab1-deficient cells, and deficient cells reconstituted with Gab1 WT or respective mutants.

Schiemann et al. have already demonstrated the impact of tyrosine 759 within gp130 on IL-6-induced Erk1/2 activation. The phosphorylated tyrosine 759-motif within gp130 is the binding site for the adapter and phosphatase SHP2. SHP2 is tyrosine-phosphorylated in response to IL-6. Phosphorylated SHP2 is suggested to bind Grb2/SOS to initiate Erk1/2 signalling [4]. Lehmann et al. showed that loss of phosphorylation of tyrosine 759 in gp130 and loss of SHP2 recruitment to gp130 completely diminishes Erk1/2 pathway activation [14]. However, IL-6 also induces SHP2 binding to Gab1 [22]. We elucidated the impact of SHP2 recruitment to gp130 or to Gab1 on SHP2 phosphorylation and Erk1/2 activation. SHP2 phosphorylation was not affected by the loss of Gab1 expression, but by mutating tyrosine 759 within gp130. Of note, tyrosine 759 within gp130 is sufficient for SHP2 phosphorylation, i.e. mutating any other tyrosine within the cytoplasmic part of gp130 does not affect SHP2 phosphorylation. Furthermore, mutating tyrosine 759 within gp130 impairs immediate and late Erk1/2 activity (Fig. 3d) whereas depletion of Gab1 mainly affects late Erk1/2 activity (Figs. 1 and 2c). Obviously, binding of SHP2 to tyrosine 759 in gp130 and Gab1 expression are crucial for efficient Erk1/2 activation in response to IL-6 signalling. We propose that initial Erk-activation is needed for initiation of Gab1-dependent signalling by recruiting Gab1 to the plasma membrane [9, 10] (Fig. 11). Actually, mutating tyrosine 759 within gp130 impairs Gab1 serine 552 phosphorylation (Fig. 5b), subsequent membrane targeting (Fig. 5a), and tyrosine phosphorylation of Gab1 (Fig. 4a, 5b). Gab1 tyrosine phosphorylation can be reconstituted by forced targeting of SHP2 to gp130 (Fig. 4b).

So far, we have no evidence for a direct interaction of Gab1 with gp130. In contrast Gab1 interacts with c-MET in response to HGF signalling [6] and with EGFR in response to EGF [7]. For EGF signalling, the signal strength dictates whether Gab1 is recruited to the EGFR (in case of strong stimulation) or to PIP3 at the plasma membrane (in case of weak stimulation) to contribute to Erk activation [7]. Thus, weak EGF stimuli and IL-6 might activate Erk1/2 through similar mechanisms. Indeed, IL-6 is a much weaker activator of Erk1/2 than EGF, or any other growth factor.

Different mechanisms of Gab1-dependent Erk1/2 activation are feasible. Montagner et al. showed that EGF-induced binding of SHP2 to Gab1 leads to dephosphorylation of the binding site for RasGAP in Gab1 [27]. As RasGAP is an inhibitor of Erk1/2 activation, dephosphorylation of its binding site in Gab1 would increase Erk1/2 activity. Additionally, the Grb2:SOS complex binds to Gab1 [28] and to Gab1-bound SHP2. In any case, Grb2:SOS is close enough to the plasma membrane to be able to activate membrane-bound Ras [5]. Here, we show for IL-6 signalling that both, binding of SHP2 to Gab1 and binding of Grb2 to Gab1 contribute to Gab1-dependent Erk1/2 activation (Figs. 6C, 7B.). Furthermore, phosphorylation of the SHP2 binding site within Gab1 and binding of SHP2 to Gab1 depends on binding of Grb2 to Gab1 (Fig. 7). So far, it remains unknown how the Grb2 binding site affects phosphorylation of the SHP2 binding site. The tyrosine kinases phosphorylating Gab proteins have not been identified, yet. However, JAKs, receptor tyrosine kinases, and Src kinases are discussed [5, 29–31]. Therefore, kinases and interactions facilitating Gab1 phosphorylation still need to be elucidated.

Our results indicate a correlation of the Gab1 expression level and the strength of Erk1/2 pathway activation (Fig. 6a). Hu et al. showed that a high Gab1 expression level correlates with epithelial ovarian cancer progression and poor prognosis [32]. Therefore, it is worth to investigate whether Gab1 expression levels correlate with progression of other solid tumour types or leukaemia in detail.

Here, we show that Gab1 influences the duration of IL-6-induced Erk1/2 pathway activation. It is well known that duration of Erk1/2 activation is critical for cell fate decision in many cellular systems [33]. E.g. platelet-derived growth factor (PDGF) induces a sustained Erk1/2 phosphorylation whereas EGF induces only a transient Erk1/2 phosphorylation. Murphy et al. showed that PDGF induced Erk1/2 activation leads to accumulation of the early gene product c-Fos, resulting in S phase entry and cell cycle progression. Here, we observed Gab1-dependent enhancement of IL-6-induced c-Fos mRNA expression in HEK293 cells and in normal human dermal fibroblasts. In contrast, EGF-induced Erk1/2 phosphorylation does not induce c-Fos expression and therefore does not promote cell cycle progression [34]. Nerve growth factor (NGF) induces sustained Erk1/2 activation and neurite outgrowth of PC12 adrenal pheochromocytoma cells. In contrast EGF initiates transient Erk1/2 phosphorylation and does not induce neurite outgrowth but proliferation [35]. PC12 cells do not respond to IL-6 alone. However, PC-12 cells pretreated with NGF respond to IL-6 with neurite outgrowth [36]. Of note, this response depends on IL-6-induced Erk1/2 activation and is counteracted by IL-6-induced STAT3 activation. Mutated IL-6-receptor complexes which do not activate STAT3 exert sustained Erk1/2 activation (probably due to the lack of SOCS3 feedback inhibition) and induce neurite outgrowth even in the absence of NGF [36]. Taken together, duration of Erk1/2 pathway activation and MAPK-dependent gene expression influence cell fate decisions in many cellular systems in response to different cytokines or growth factors. Gab1 determines the duration of IL-6-induced Erk1/2 pathway activation by amplification of this signal transduction pathway. Therefore, it is likely that dysregulated Gab1 significantly impacts on cell fate decisions and may contribute to cancer progression. For example, the uncontrolled proliferation of Jak2-V617F positive leukaemia cells might thus be explained by malregulated Gab1 and constitutive activation of Erk1/2 as observed in Jak2-V617F-positve cells [11, 12].

## Conclusions

We elaborated the molecular requirements for Gab1-dependent orchestration of interleukin-6-dependent MAPK signalling. We discriminated IL-6-induced Gab1-independent, early activation of MAPK signalling and Gab1-dependent, sustained activation of MAPK signalling. This study underlines the impact of SHP2 binding to gp130 for early Erk1/2 activation and the impact of binding of SHP2 and Grb2 to Gab1 for sustained Erk1/2 activation.

## Data Availability

The datasets used and/or analysed during the current study are available from the corresponding author on reasonable request.

## Abbreviations

AEBSF: 4-(2-Aminoethyl)-benzolsulfonylfluorid
DMEM: Dulbecco’s modified Eagle’s medium
Dox: doxycycline
EGF: epidermal growth factor
Epo: Erythropoietin
EpoR: erythropoietin receptor
Erk: extracellular signal regulated kinase
FCS: fetal calf serum
Gab1: Grb2-associated binder 1
Grb2: growth factor receptor bound protein 2
HEK: human embryonic kidney
HGF: hepatocyte growth factor
IL-5: interleukin-5
IL-6: interleukin-6
Jak: Janus Kinase
MAPK: mitogen activated protein kinase
MEF: murine embryonic fibroblasts
NGF: nerve growth factor
NHDF: normal human dermal fibroblasts
PDGF: platelet-derived growth factor
PH: pleckstrin homology
PI3K: phosphatidyl-inositol-3-kinase
PIP3: phosphatidylinositol (3:4:5)-trisphosphate
PKB/Akt: protein kinase B
PLC: phospholipase C
RasGAP: RasGTPase activating protein
SDHA: succinate dehydrogenase complex flavoprotein subunit A
SHP2: Src-homology domain containing phosphatase
STAT: signal transducer and activator of transcription
